# A Risk Model based on Ultrasound, Ultrasound Elastography, and Histologic Parameters for Predicting Axillary Lymph Node Metastasis in Breast Invasive Ductal Carcinoma

**DOI:** 10.1038/s41598-017-03582-3

**Published:** 2017-06-08

**Authors:** Xiao-Long Li, Hui-Xiong Xu, Dan-Dan Li, Ya-Ping He, Wen-Wen Yue, Jun-Mei Xu, Bo-Ji Liu, Li-Ping Sun, Lin Fang

**Affiliations:** 1 Department of Medical Ultrasound, Shanghai Tenth People’s Hospital, Ultrasound Research and Education Institute, Tongji University School of Medicine, Shanghai, 200072 China; 2 Department of Thyroid and Breast Surgery, Shanghai Tenth People’s Hospital, Tongji University School of Medicine, Shanghai, 200072 China

## Abstract

To develop a risk model for predicting axillary lymph node metastasis (LNM) in patients with breast invasive ductal carcinoma (IDCs) using ultrasound (US), US elastography of virtual touch tissue imaging (VTI) and virtual touch tissue imaging & quantification (VTIQ), and histologic parameters. This study included 162 breast IDCs in 162 patients. Univariate and multivariate analyses were used to identify the risk factors and a risk model was created. The results found that 64 (39.5%) of 162 patients had axillary LNMs. The risk score (RS) for axillary LNM was defined as following: RS = 1.3 × (if lesion size ≥20 mm) + 2.6 × (if taller than wide shape) + 2.2 × (if VTI score ≥5) + 3.9 × (if histological grade III) + 1.9 × (if positive C-erbB-2). The rating system was divided into 6 stages (i.e. Stage I, Stage II, Stage III, Stage IV, Stage V, and Stage VI) and the associated risk rates in terms of axillary LNM were 0% (0/19), 6.1% (2/33), 7.7% (3/39), 65.5% (19/29), 92.3% (24/26), and 100% (16/16), respectively. The risk model for axillary LNM established in the study may facilitate subsequent treatment planning and management in patients with breast IDCs.

## Introduction

Breast cancer has become a global health problem and caused more and more death in females in recent years. Invasive ductal carcinoma (IDC) is the most common breast cancer of women^1^. Axillary lymph node is the most common site of metastasis for breast cancer and it is an extremely important factor when making treatment decision^2^. Therefore, how to detect axillary lymph node metastasis (LNM) of breast cancer at an early stage is becoming a crucial issue. Traditionally, patients are subject to axillary lymph node dissection (ALND) to evaluate LNM. However, most axillary lymph nodes are normal and the value of ALND operation is still uncertain. In the past few years, several studies have reported that survival is not affected by clearance of regional lymph nodes^3, 4^. In many centers, sentinel node biopsy (SLNB) as a minimally invasive modality has largely replaced ALND for evaluation of the axilla^5–7^. However, SLNB is dependent on the operator’ s experience and may have false negative results^2–4^. In cases of several drawbacks and ineligibility of surgical method, a non-surgical method to predict axillary LNM is needed for drawing up a plan of systemic therapy, thus clinical management of patients could be improved.

Conventional high frequency ultrasound (US) is commonly used to detect breast lesion. Various studies have showed that conventional US may be helpful for predicting axillary LNM by evaluating the suspicious US features such as intralesional calcification, internal flow, and nodal size^8, 9^. On the other hand, many studies have showed that breast cancers are stiffer than normal and benign tissues^10–12^, which can be evaluated by US elastography. As new US-based elastography techniques, acoustic radiation force impulse (ARFI) imaging of virtual touch tissue imaging (VTI; Siemens Medical Solutions, Mountain View, CA, USA) and shear wave imaging of virtual touch tissue imaging & quantification (VTIQ; Siemens Medical Solutions, Mountain View, CA, USA) have recently been used to diagnose breast cancers by measurement of the tissue stiffness^10, 11^. For both VTI and VTIQ, a short-duration acoustic pulse from the transducer is used to generate vertical pressure and lateral local displacement. VTI is a qualitative method whereas VTIQ is a quantitative method. The latter reflects the tissue stiffness through the measurement of shear wave speed (SWS)^13^. The maximum depth of generating sufficient shear waves (SWs) is approximately 4.5 cm because of the attenuation of ARFI^14^.

Although shear wave imaging such as Supersonic imagine (SSI, Aix en Provence, France) has been used to evaluate axillary LNM in patients with breast IDCs^15^, until present no study has been carried out using VTI and VTIQ for predicting axillary LNM. We hypothesized that VTI and VTIQ might be useful for predicting axillary LNM. What is more, histological grade was also used as a prognostic factor to assess patient survival and disease recurrence^16^. On the other hand, estrogen receptor (ER), progesterone receptor (PR), and C-erbB-2 (i.e. human epidermal growth factor receptor 2 [HER 2]) expression are the most important prognostic factors for management of breast cancer, particularly in metastatic disease settings and adjuvant treatment. Thus, it was considered that the effect of the prognostic parameters including histological grade, ER, PR, and C-erbB-2 expression on axillary LNM of IDC patient should be taken into account. The purpose of the present study was to identify the risk factors including conventional US, VTI, VTIQ, and the histologic parameters and then to propose a new risk model for predicting axillary LNM in patients with breast IDCs.

## Materials and Methods

The retrospective study was approved by the Ethical Committee of the Shanghai Tenth People’s Hospital of Tongji University School of Medicine and informed consent from the patients were waived. The study was performed in accordance with relevant guidelines and regulations.

### Patients

From June 2014 to September 2015, a total of 174 consecutive women were enrolled in the study. All the patients were referred to examination by conventional US, VTI, and VTIQ for the evaluation of breast lesions that were detected incidentally during a prior imaging study or had been discovered with clinical palpation. The enrollment criteria for the patients were as follows: (1) breast IDC confirmed by histopathology after surgery; (2) solid or almost solid (<25% cystic) breast lesion with size larger than 5 mm in diameter and depth ≤ 4.5 cm on conventional US; and (3) patient without any previous treatment. Twelve patients with incomplete preoperative US, VTI and VTIQ data were excluded. For patients with multiple lesions (nine patients had two lesions and seven patients had three or even more lesions), only the largest one in diameter on US was included. Eventually, 162 patients (mean age, 57.2 years ± 11.4; age range, 26–87 years) with 162 lesions comprised the final study cohort. Among them, 37 of 162 IDCs had been reported in a previous study with an aim to evaluate the value of VTIQ for US Breast Imaging Reporting and Data System (BI-RADS) category 4 lesions^13^.

### Conventional US, VTI, and VTIQ

Conventional US, VTI, and VTIQ were performed with the same Siemens S3000 US scanner (Siemens Medical Solutions, Mountain View, CA, USA). Conventional US, VTI, and VTIQ were performed by one of three skillful radiologists who had 2 years’ experience in breast US imaging and 2 years’ experience in VTI and VTIQ imaging. Each lesion was scanned with the patient holding their breath for a few seconds in the supine position. For conventional US, a 18L6 linear array transducer (frequency range, 6–18 MHz) was used based on the American Institute of Ultrasound in Medicine practice guideline^17^.

VTI and VTIQ were performed following conventional US using the 9L4 linear transducer (frequency range, 4.0–9.0 MHz). The scanning was performed in the longitudinal plane of the lesion and pre-compression were avoided. The VTI imaging reflects the tissue elasticity with two grayscale values (i.e. black and white) in the field of view (FOV), which includes the lesion and some surrounding breast tissues. The gray scale value of black indicates hard tissue and the white indicates soft tissue. VTIQ measurement was then performed to measure SWS within the breast lesion. The measurement result of SWS is expressed as “m/s” and ranges from 0.5 to 10 m/s^18^. VTIQ imaging involves the target lesion and sufficient surrounding tissue as well. The SW-quality map was firstly applied, which displayed quality in different colors from high (green), moderate (yellow), to low (red) quality^19^. SWS measurement was then performed on SW-velocity map by placing the region of interest (ROI) on the areas with high SW quality in the breast lesion. In SW-velocity map, the speed distribution is shown in different colors from high (red), intermediate (yellow or green), to low (blue). The scale of the SW-velocity map is set at default value of 10 m/s and is not adjusted thereafter. Seven SWS measurements were performed, and the cystic or calcified areas were avoided. The smallest size of ROI box is around 2 × 2 mm. The ROIs were placed at random when the distribution of lesion stiffness was homogeneous. Otherwise two ROIs were placed on the highest stiffness area and the lowest stiffness area respectively, and the remaining five ROIs were placed at random. The mean SWS values were computed. All dates from conventional US, VTI, and VTIQ were recorded for further analysis.

### Image Analysis

Two independent investigators were asked to analyze all the US, VTI, and VTIQ images in a blind manner. Both investigators had more than 3 years of experience in breast US. Disagreement was solved by consensus. Each investigator had been trained to review the images before the study. The conventional US features as following were evaluated: lesion size (≥ 20 mm/< 20 mm), shape (regular/irregular), margin (circumscribed/not circumscribed), inhomogeneous echo, posterior features (absent/present), microcalcifications, taller than wide or not, and internal flow on color Doppler US (poor/rich). The calcification was judged to be microcalcification if the calcification diameter ≤0.5 mm^20^. In addition, the vascularity of the lesions were determined as described by Xu *et al*.^21^. The internal flow was judged to be “rich” if more than 3 linear or tree-like signals existed on color Doppler US, otherwise the flow was judged to be “poor” (i.e. dotted signals or without signals on color Doppler US). The VTI of the breast lesions was scored from 1 (soft) to 6 (hard) as described by Li *et al*.^13^: score of 1, predominantly white; score of 2, predominantly white with few black portions; score of 3, black and white portion equally; score of 4, predominantly black with a few white spots; score of 5, almost completely black; and score of 6, completely black.

### Histopathological Analysis

Pathological reports after surgeries were reviewed to determine histological grade of the tumor, presence of axillary LNM or not, and the number of the metastatic lymph node status. The histologic grade for each lesion was recorded as grade I, grade II, or grade III (Elston and Ellis method)^16^. We regarded the grade I, grade II as “low” and grade III as “high”. The routine paraffin sections was used as the reference standard and the immunohistochemical staining were then used to get the results such as ER, PR, and C-erbB-2 expression. Briefly, four micrometer paraffin sections were cut and air dried overnight at room temperature (temperature: <25 °C). After deparaffinization with xylene and hydration in decreasing grades of ethanol, the activity of endogenous peroxidase was eliminated by treating the slides with a solution of hydrogen peroxide and methanol. The pressure cooker method in 0.1 mol/l sodium citrate buffer (pH 6) was used to retrieve heat-induced epitope. Then the slides were treated with the diluted primary antibody, biotinylated antirabbit IgG immunoglobulin, and streptavidinperoxidase complex. Diaminobenzidine was used as a chromogen in the presence of hydrogen peroxide (View Universal DAB Detection Kit, Ventana®, Roche, Switzerland). Slides were sequentially counterstained with Mayer’s haematoxylin, dehydrated in alcohol, cleared in xylene and mounted. The ER and PR were judged to be positive if the expression was ≥10%. The C-erbB-2 expression was scored as negative, 1+, 2+, and 3+. We judged the C-erbB-2 expression to be positive if the expression was 2+ or 3+^22^, otherwise C-erbB-2 expression was judged to be negative.

### Statistical Analysis

Statistical analyses were performed with SPSS software (version 20.0 for Windows; SPSS, Chicago, IL, USA). Quantitative datas (i.e. patient age, lesion size, and SWS on VTIQ) were expressed as mean ± standard deviation (SD) if normal distribution was achieved. Continuous variables were compared by independent t test. Categorical variables were analyzed by χ^2^ test or Fisher’s exact probability test. The best cut-off value for VTI was obtained to differentiate between patients with axillary LNM and no axillary LNM. The correlation between the predicting factors and axillary LNM was analyzed by univariate analysis. Multivariate logistic regression analysis was used to determine the statistically significant factors. Odd ratios (ORs) with 95% confidence interval (CI) were recorded and the equation for predicting axillary LNM was created. Receiver operating characteristic (ROC) curves were plotted to assess the diagnostic performance of the statistically significant factors and the predictive equation. The diagnostic performances for the statistically significant factors and the predictive equation were expressed as the area under the ROC curve (AUROC). The diagnostic performance was shown in different AUROC from low (AUROC = 0.5–0.7), moderate (AUROC = 0.7–0.9), to high (AUROC > 0.9). ROC analysis was used to assess the sensitivity and specificity. The best cut-off values for the predictive equation and VTI were obtained when Youden indexs (YIs) were maximum (sensitivity + specificity − 1). *P* < 0.05 was considered as statistical significance.

## Results

**Figure 1:**
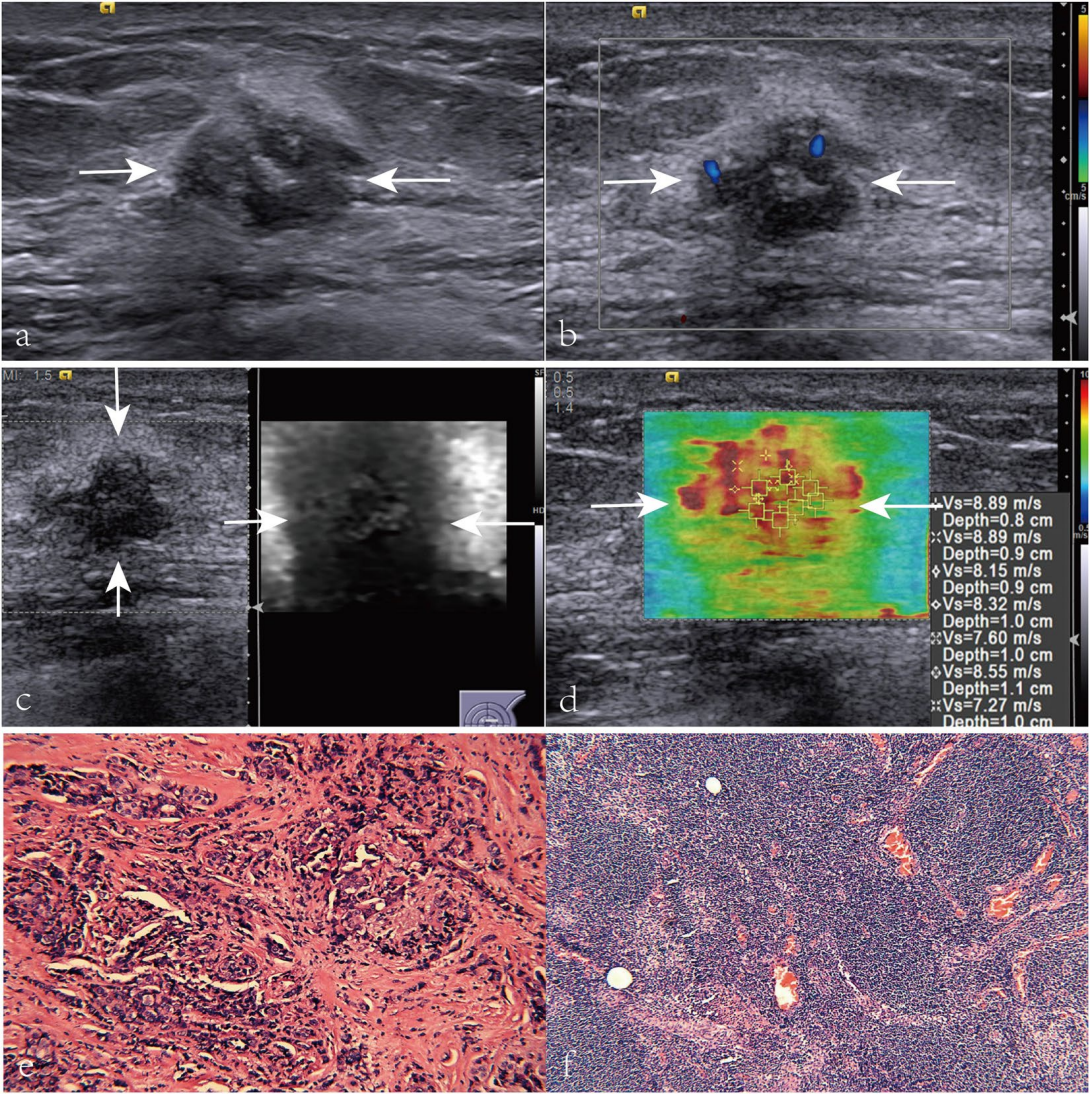
Images in a 60-year-old patient with breast invasive ductal carcinoma, no axillary lymph node metastasis (LNM), histologic grade II, positive estrogen receptor (ER), negative progesterone receptor (PR), and negative C-erbB-2. (**a**) A solid, heterogeneous hypoechogenicity, and poorly defined margin lesion (arrows) is shown on ultrasound (US). (**b**) Poor internal flow (i.e. dotted signals on color Doppler US) is found on color Doppler flow image (arrows). (**c**) Virtual touch tissue imaging (VTI) score of the lesion (arrows) is 3. (**d**) On virtual touch tissue imaging & quantification image, the lesion (arrows) is heterogeneous with a mean SWS value of 8.24 m/s. (**e**) Pathological examination confirms the diagnosis of invasive ductal carcinoma (Hematoxylin-eosin stain, x200). (**f**) Pathological examination confirms the diagnosis of normal lymph node (Hematoxylin-eosin stain, x200).

**Figure 2:**
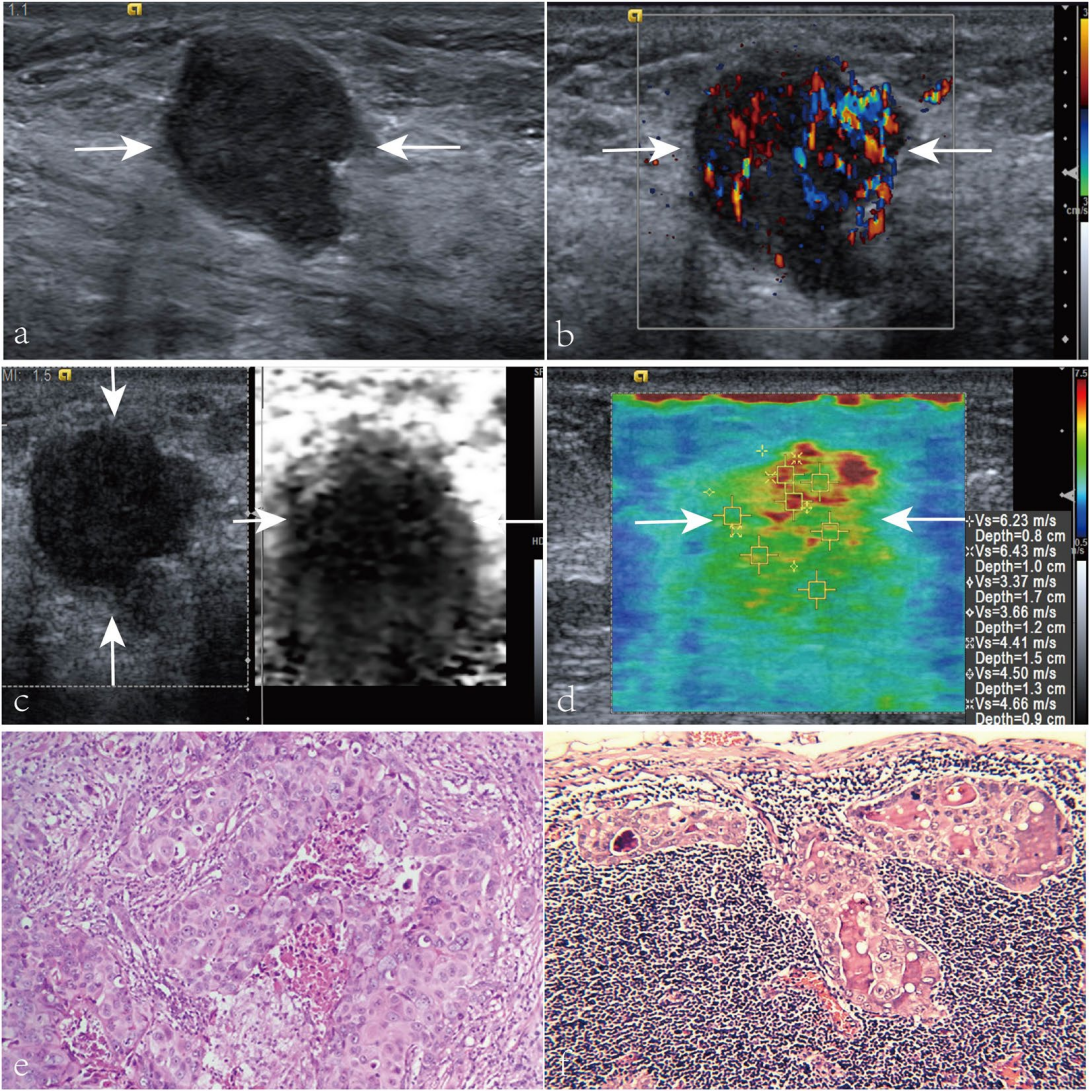
Images in a 58-year-old patient with breast invasive ductal carcinoma, axillary lymph node metastasis (LNM), histologic grade III, negative estrogen receptor (ER), negative progesterone receptor (PR), and positive C-erbB-2. (**a**) A solid, marked hypoechogenicity, well defined margin, irregular, and taller than wide shape lesion (arrows) is shown on US. (**b**) Rich internal flow (i.e. 3 linear or tree-like signals) is found on color Doppler flow image (arrows) of the breast invasive ductal carcinoma. (**c**) Virtual touch tissue imaging (VTI) score of the lesion (arrows) is 4. (**d**) On virtual touch tissue imaging & quantification image, the lesion (arrows) is heterogeneous with a mean SWS value of 4.75 m/s. (**e**) Pathological examination confirms the diagnosis of invasive ductal carcinoma (Hematoxylin-eosin stain, ×200). (**f**) Pathological examination confirms the diagnosis of axillary LNM (Hematoxylin-eosin stain, ×200).

Of the 162 breast lesions, the final pathological diagnoses were all IDCs. Among them, 20 (12.3%) were histologic grade I, 84 (51.9%) were histologic grade II, and 58 (35.8%) were histologic grade III. Axillary LNMs were present in 64 (39.5%) patients whereas absent in 98 (60.5%) patients (Figs 1 and 2). Number of metastatic lymph nodes were three or even more in 24 (37.5%) patients and one or two in 40 (62.5%) patients.

The mean size of the breast lesions was 26.6 mm ± 15.4 (range, 6.0–86.0 mm). The breast lesions with metastatic lymph nodes tended to be larger than those without metastatic lymph nodes (31.2 ± 16.0 mm; range, 9.0–84.0 mm vs. 23.4 ± 15.3 mm; range, 6.0–86.0 mm) (*P* = 0.002). The basic characteristics and US features of the patients and lesions are listed in Table 1.

**Table 1:** Basic characteristics and ultrasound features of the patients.

Characteristics	Axillary LNM		*P* value
	Yes	No	
Mean age (y, range)	56.16 ± 9.43 (34–81)	57.87 ± 12.52 (26–87)	0.352
Lesion size ( mm, range)	31.16 ± 16.02 (9–84)	23.43 ± 15.30 (6–86)	0.002*
Lesion position			0.599
Right/left, n (%)	28 (43.8)/36 (56.2)	47 (48.0)/51 (52.0)	
Lesion shape			0.368
Regular/irregular, n (%)	5 (7.8)/59 (92.2)	12 (12.2)/86 (87.8)	
Margin			0.616
Circumscribed/not circumscribed, n (%)	22 (34.4)/42 (65.6)	30 (30.6)/68 (69.4)	
Homogeneous echo			0.536
Yes/no, n (%)	13 (20.3)/51 (79.7)	24 (24.5)/74 (75.5)	
Posterior acoustic features			0.273
Absent/present, n (%)	37 (57.8)/27 (42.2)	65 (66.3)/33 (33.7)	
Microcalcifications in a lesion			0.241
Absent/present, n (%)	38 (38.8)/26 (26.5)	67 (68.4)/31 (31.6)	
Taller than wide shape			<0.001*
No/yes, n (%)	14 (21.9)/50 (78.1)	65 (66.3)/33 (33.7)	
Internal flow			<0.001*
Poor/rich, n (%)	22 (34.4)/42 (65.6)	73 (74.5)/25 (25.5)	

LNM lymph node metastasis. *Indicates statistically significant difference.

**Figure 3:**
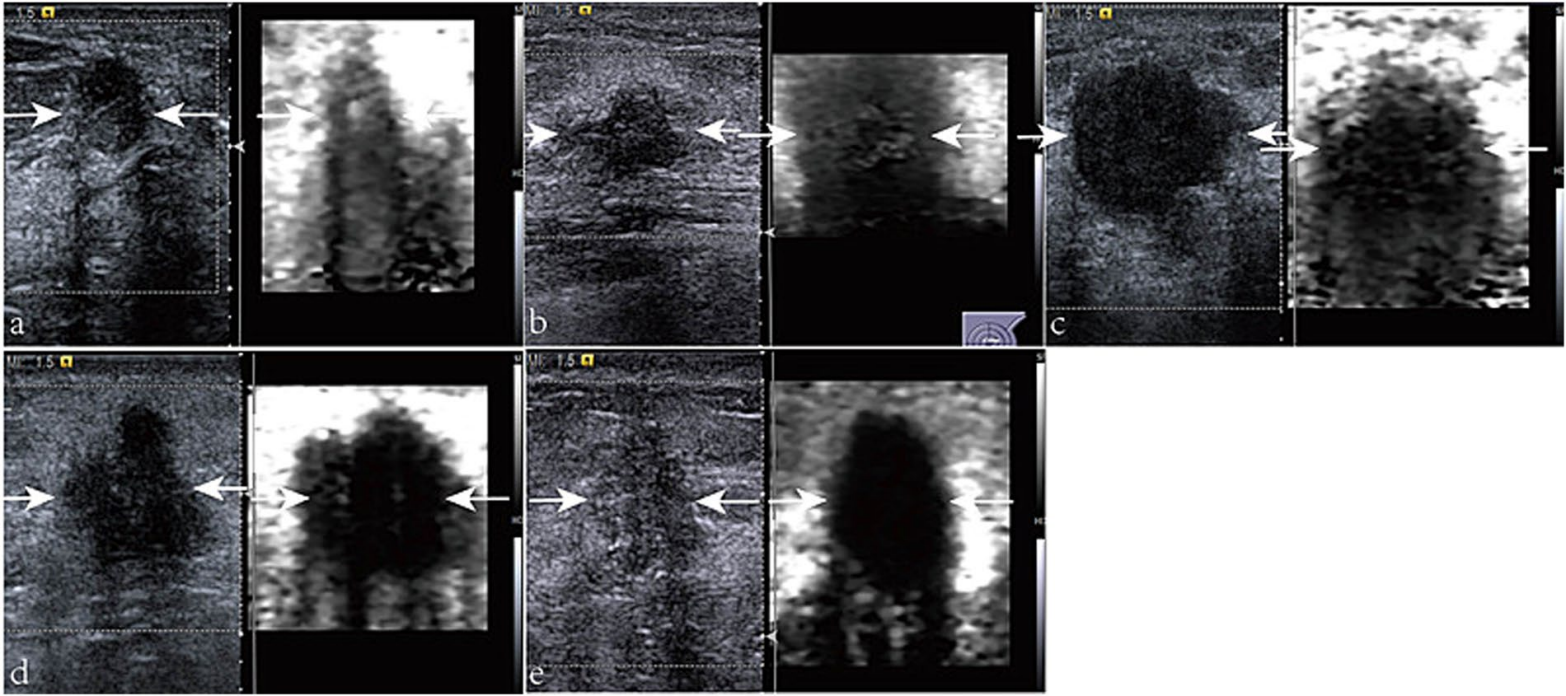
Virtual Touch tissue imaging elasticity scores of the lesions (arrows): (**a**) score 2; (**b**) score 3; (**c**) score 4; (**d**) score 5; and (**e**) score 6.

Comparisons between patients with axillary LNM and without axillary LNM in terms of VTI, VTIQ, and histologic parameters are listed in Table 2. For VTI, tissue stiffness of the breast lesions (Fig. 3) was scored from 2 to 6 and the best cut-off value was VTI score ≥5 (YI = 0.470). No axillary LNM was found for those patients with VTI score of 1 and 2 lesions. were found Axillary LNM was found more frequently in patients with VTI scores of 5 and 6 lesions than in those with VTI scores of 3 and 4 lesions (*P* < 0.001). The sensitivity and specificity for VTI were 76.6% (49/64) and 70.4% (69/98) respectively.

**Table 2:** Comparisons with VTI, VTIQ, and histologic parameters between breast lesions with axillary LNM and those without axillary LNM.

Parameters	Overall lesions (n = 162)	Axillary lymph node metastasis		*P* value
		Yes (n = 64)	No (n = 98)	
VTI				<0.001*
Score 1	0 (0.0%)	0 (0.0%)	0 (0.0%)	
Score 2	3 (1.9%)	0 (0.0%)	3 (3.1%)	
Score 3	15 (9.3%)	3 (4.7%)	12 (12.2%)	
Score 4	66 (40.7%)	12 (18.8%)	54 (55.1%)	
Score 5	72 (44.4%)	45 (70.3%)	27 (27.6%)	
Score 6	6 (3.7%)	4 (6.3%)	2 (2.0%)	
VTIQ				0.651
SWS (m/s)	5.60 ± 1.62	5.53 ± 1.52	5.64 ± 1.68	
Range (m/s)	3.01–9.68	3.01–9.22	3.12–9.68	
Histologic grade				<0.001*
I	20	19	1	
II	84	69	15	
III	58	10	48	
ER status				0.716
Negative	48	20	28	
Positive	114	44	70	
PR status				0.306
Negative	99	36	63	
Positive	63	28	35	
C-erbB-2 expression				<0.001*
Negative	79	16	63	
Positive	83	48	35	

VTI virtual touch tissue imaging, VTIQ virtual touch tissue imaging & quantification, LNM lymph node metastasis, SWS shear wave speed, ER estrogen receptor, PR progesterone receptor. *Indicates statistically significant difference

**Figure 4:**
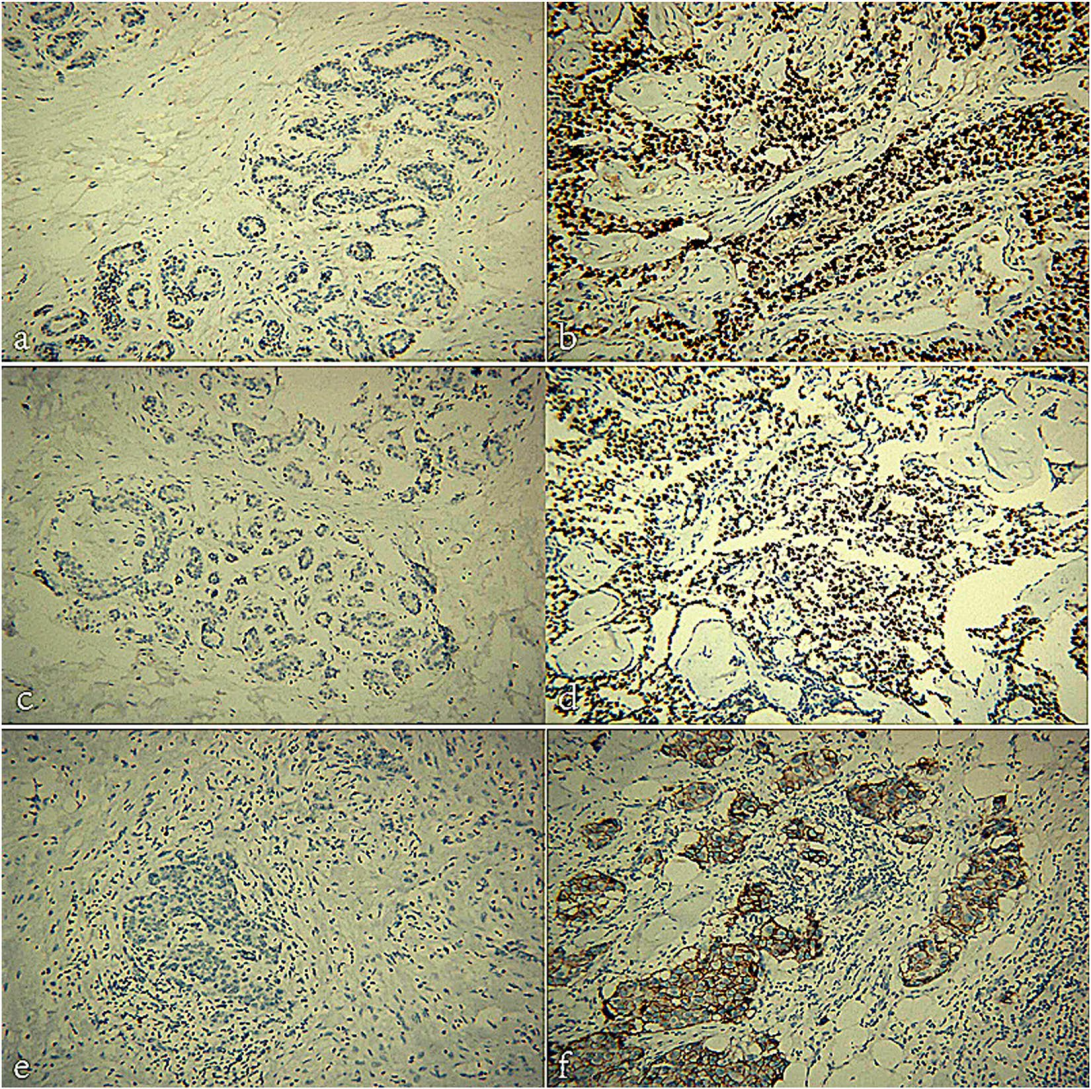
Representative immunohistochemical staining of estrogen receptor (ER), progesterone receptor (PR), and C-erbB-2. (**a**) ER status (negative); (**b**) ER status (positive); (**c**) PR status (negative); (**d**) PR status (positive); (**e**) C-erbB-2 expression (negative); and (**f**) C-erbB-2 expression (positive).

In addition, the immunohistochemical results such as ER, PR, and C-erbB-2 expression (Fig. 4) were obtained. Metastatic lymph nodes were found in 38.6% (44/114) patients with positive ER (expression ≥10%) and 41.7% (20/48) patients with negative ER (expression <10%). On the other hand, metastatic lymph nodes were found in 44.4% (28/63) patients with positive PR (expression ≥10%) and 36.4% (36/99) patients with negative PR (expression <10%). However, there was no statistical differences between patients with positive and negative ER in terms of axillary LNM, as well as those with positive and negative PR (both *P* > 0.05). For C-erbB-2 expression, metastatic lymph nodes was found in 57.8% (48/83) patients with positive expression (2+ or 3+) and 20.3% (16/79) patients with negative expression (negative or 1+). Axillary LNMs were found more frequently in the patients with positive expression of C-erbB-2 (*P* < 0.001).

Univariate analysis showed that lesion size ≥20 mm, taller than wide shape, rich internal flow on color Doppler US, VTI score ≥5, histologic grade III, and positive C-erbB-2 were significantly associated with axillary LNM (all *P* < 0.05) (Table 3). None of the factors such as age, margin, shape, microcalcification, homogeneous echo, posterior features, SWS value on VTIQ, ER, and PR were found to be statistically significant in predicting axillary LNM in patients with breast IDCs (all *P* > 0.05).

**Table 3:** Univariate analysis in predicting axillary lymph node metastasis.

Parameters	Odds Ratios	95% CI	*P*
Lesion size ≥20 mm	0.334	2.321–9.115	<0.001*
Taller than wide shape	0.215	3.404–14.536	<0.001*
Rich internal flow	5.575	2.804–11.084	<0.001*
VTI score ≥5	7.772	3.772–16.015	<0.001*
Histologic grade III	0.182	11.118–62.698	<0.001*
Positive C-erbB-2	5.400	2.680–10.881	<0.001*

CI confidence interval. *Indicates statistically significant difference.

On multivariate logistic regression analysis, all independent predictors were determined as following: lesion size ≥20 mm (OR: 3.526, *P* = 0.033), taller than wide shape (OR: 14.102, *P* < 0.001), VTI score ≥5 (OR: 9.003, *P* < 0.001), histological grade III (OR: 49.127, *P* < 0.001), and positive C-erbB-2 (OR: 6.869, *P* = 0.002) (Table 4). Among them, histological grade III was the most significant risk factor.

**Table 4:** Multivariate analysis in predicting axillary lymph node metastasis.

Parameters	B	SE	Odds Ratios	95% CI	*P*
Lesion size ≥20 mm	1.260	0.592	3.526	1.104–11.259	0.033*
Taller than wide shape	2.646	0.715	14.102	3.471–57.292	<0.001*
VTI score ≥5	2.198	0.620	9.003	2.671–30.345	<0.001*
Histologic grade III	3.894	0.759	49.127	11.100–217.418	<0.001*
Positive C-erbB-2	1.927	0.608	6.869	2.088–22.600	0.002*

VTI virtual touch tissue imaging, SE standard error, CI confidence interval. *Indicates statistically significant difference.

**Figure 5:**
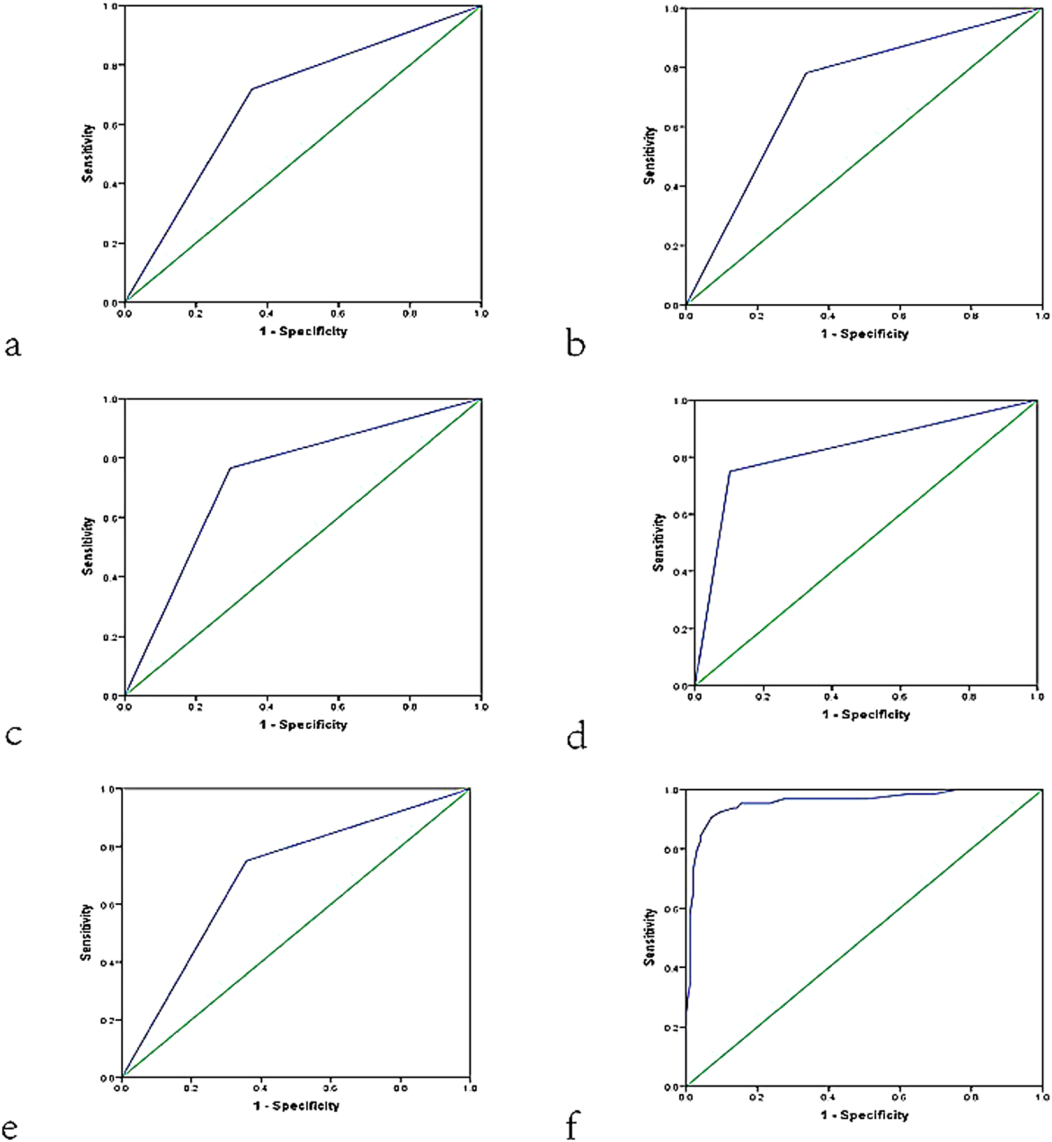
Receiver operating characteristic (ROC) curves of (**a**) the lesion size ≥20 mm (AUROC = 0.681), (**b**) taller than wide shape (AUROC = 0.722), (**c**) VTI score ≥5 (AUROC = 0.735), (**d**) histological grade III (AUROC = 0.824), (**e**) C-erbB-2 positive (AUROC = 0.696), and (**f**) the predictive equation (AUROC = 0.958) for prediction of axillary lymph node metastasis (LNM).

A multivariate logistic regression equation was then established with the significant predictors as follows: P = 1/1 + Exp∑[−6.317 + 1.260 × (if lesion size ≥20 mm) + 2.646 × (if taller than wide shape) + 2.198 × (if VTI ≥5) + 3.894 × (if histological grade III) + 1.927 × (if positive C-erbB-2)]. Then the ROC curves were plotted to assess the diagnostic performances of the predictive equation, lesion size ≥20 mm, taller than wide shape, VTI score ≥5, histological grade III, and positive C-erbB-2. The diagnostic values of the predictive equation, lesion size ≥20 mm, taller than wide shape, VTI score ≥5, histological grade III, and positive C-erbB-2 were 0.958, 0.681, 0.722, 0.735, 0.824, and 0.696, respectively (Fig. 5). In terms of AUROC, the predictive equation achieved the highest diagnostic performance. The best cut-off value for the predictive equation was −0.22 (YI = 0.835). The sensitivity and specificity were 90.6% and 92.9% respectively.

A final predicting model was established based on the 5 risk factors derived from the multivariate logistic regression analysis. Risk score (RS) for each lesion was defined as follows: RS = 1.3 × (if lesion size ≥20 mm) + 2.6 × (if taller than wide shape) + 2.2 × (if VTI score ≥5) + 3.9 × (if histological grade III) + 1.9 × (if positive C-erbB-2). The rating system was divided into 6 stages as following: Stage I, RS was <1.3 and none of 5 risk factors was enrolled, including 19 (11.7%) patients; Stage II, RS was 1.4 to 3.9 and any 1 of 5 risk factors was enrolled, including 33 (20.4%) patients; Stage III, RS was 4.0 to 6.5 and any 2 of 5 risk factors were enrolled, including 39 (24.1%) patients; Stage IV, RS was 6.6 to 8.7 and any 3 of 5 risk factors were enrolled, included 29 (17.9%) patients; Stage V, RS was 8.8 to 10.6 and any 4 of 5 risk factors were enrolled, including 26 (16.0%) patients; and Stage VI, RS was 10.7 to 11.9 and all of 5 risk factors were enrolled, including 16 (9.9%) patients. The risk rates of axillary LNM were 0% (0/19) in Stage I, 6.1% (2/33) in Stage II, 7.7% (3/39) in Stage III, 65.5% (19/29) in Stage IV, 92.3% (24/26) in Stage V, and 100% (16/16) in Stage VI. According to the results of the rating system, as the number of risk factors increased, the probability of axillary LNM increased. With these findings, we regarded Stage I (none risk factors) as no axillary LNM probably, Stage II and Stage III (one or two risk factors) as low suspicion, Stage IV (three risk factors) as mediate suspicion, and Stage V and Stage VI (four or five factors) as highly suggestive of axillary LNM.

## Discussion

Traditionally, ALND can remove the lymph node and reduce the concern for LNM. However, ALND had some drawbacks such as lymphedema, pain, or shoulder weakness^4, 23^. Therefore, SLNB has become a safe method to replace ALND. Susan *et al*. found that the sensitivity of US-guided FNA for predicting positive results was 71–75%^24^. However, the obvious disadvantage was that the sensitivity increased with increasing size of the primary lesion. Several studies showed that the false negative results would never reach zero^2, 4, 25^. What is more, it is generally known that conventional US is wildly used to find abnormal lymph node by examination of axilla. However, not all metastatic lymph nodes could be seen by conventional US. Several studies have reported that metastatic lymph nodes were seen in only 35–65% of the patients by conventional US^2, 4, 26^. This is probably a consequence of the latency metastasis, which may not cause the abnormity of lymph node on conventional US^27^. As shown in the present study, the predicting model based on US, US elastography, and histologic parameters, as a noninvasive method, achieved the high diagnostic performance (AUROC = 0.958). Therefore, it may be a useful method to predict axillary LNM.

In the present study, seventeen clinical characteristics, US features, VTI features, VTIQ features, and histologic parameters were included as potential predictors for axillary LNM. In the past few years, several studies showed that some clinical characteristics and conventional US features were associated with axillary LNM^28, 29^. In the present study, age, shape, margin, homogeneous echo, posterior acoustic features, microcalcifications, SWS, ER, and PR were not identified to be predictors for axillary LNM. In univariate analyses, lesion size ≥20 mm was associated with axillary LNM, which was in consistent with previous studies^27, 30^. In addition, rich internal flow (OR: 5.575, *P* < 0.001) was significantly associated with axillary LNM in univariate analysis. A study by Mehta *et al*. also showed that flow of breast cancer correlated strongly with detection of lymph node involvement, with an associated sensitivity of 93%^29^. As we all know, the lymphatic drainage of breast is rich. The tumor cells may spread through the drainages. However, in multivariate logistic analysis, rich internal flow was not a independent risk factor after adjustment for other factors. What is more, the result of our study indicated that taller than wide shape (OR: 14.102, *P* < 0.001) was also associated with axillary LNM. Although Stavors *et al*. reported that the taller than wide shape was a critical factor to distinguish between benign and malignant breast lesions^31^, it has not been reported before as an independent predictive factor of axillary LNM. The morphological change of IDCs reflects the enhancement of the tumor invasion ability to some extent, which may lead to cell growth, motility, and differentiation.

Recently, tissue stiffness reflected by conventional US strain elastography (SE), strain ratio on elastography, or virtual touch tissue quantification (VTQ) imaging has been found to be associated with LNM^32–35^. However, these techniques have several limitations. SE and strain ratio on elastography have poor reproducibility and lack of quantitative information. For VTQ, the ROI for interrogation is fixed (i.e. 6 × 5 mm) and the measurement range (i.e. 0.5–8.4 m/s) is limited.

Evans *et al*. reported that metastatic lymph nodal involvement rates increased from 7% to 41% with the tumor stiffness increased from mean stiffness <50 KPa to >150 KPa by measuring tumor stiffness quantitatively^15^. VTI and VTIQ, as the latest development of elastography techniques, have been used to differentiate between benign and malignant breast lesions^13, 19^, whereas their role in predicting axillary LNM is unknown. According to our results, VTI score ≥5 had an approximately nine times (OR: 9.003) higher potential of axillary LNM than lesions with VTI score <5. Several *in vitro* studies reported that pathogenesis of tumor invasion and metastasis increased stiffness of matrix, which was an important microenvironment cue to regulate cell growth, motility, and differentiation^36–38^. On the other hand, VTIQ was not an independent risk factor for predicting axillary LNM, which was in contrast to the study by Evans *et al*.^15^. The reason may be that different sampling methods were used. In the present study, mean SWS value of the 7 measurements on each lesion was computed, while the ROI covered the entire lesion in the study by Evans *et al*. with Supersonic imagine (SSI, Aix en Provence, France). Maybe the latter measuring method reflects the whole tissue stiffness and is a better choice.

Our result demonstrated that ER and PR were not associated with axillary LNM, which was similar with the study by Evans *et al*.^15^. ER and PR might play an important role in determining whether a patient needed adjuvant endocrine therapy^39^. Histologic grade III had an approximately forty nine times (OR: 49.127) higher potential of axillary LNM than lesions with grade I or grade II. The enhanced tumor invasion ability may be the reason. What is more, in the present study, positive C-erbB-2 was an independent risk factor for predicting axillary LNM, which has been confirmed in several studies^40, 41^. The phenomenon could be correlated with an enhanced tumor aggressiveness.

A multivariate logistic regression equation was established in the present study. The result of the study suggested that the presence of axillary LNM was depending on the features such as lesion size ≥20 mm, taller than wide shape, VTI score ≥5, histological grade III, and positive C-erbB-2. The reliability of the final predicting model was confirmed by AUROC of 0.958, which was higher than that of lesion size ≥20 mm (AUROC = 0.681), taller than wide shape (AUROC = 0.722), VTI score ≥5 (AUROC = 0.735), histological grade III (AUROC = 0.824), and positive C-erbB-2 (AUROC = 0.696) individually. A risk model with six stages (i.e. Stage I, Stage II, Stage III, Stage IV, Stage V, and Stage VI) was established according to the five independent risk factors, and the corresponding risks of axillary LNM were 0%, 6.1%, 7.7%, 65.5%, 92.9%, and 100%, respectively. The results indicated that none of five predictive factors in patients at Stage I was not expected to undergo ALND. For Stage II and Stage III patients, axillary LNM was probably low, and combining other risk factors such as rich internal flow, poorly defined margin reported by previous studies might be helpful to determine the strategy of ALND^28, 29^. For Stage IV, Stage V, and Stage VI patients, we would advocate the patients to determine the strategy of ALND. Thus, based on the model, ALND as a aspiring strategy was suggested in patients at Stage IV, Stage V, and Stage VI.

The current study had several limitations. First, this was a retrospective single-centre study, which might cause selection bias. Second, breast lesions with size <5 mm in diameter were not included in the present study because the smallest size of VTIQ ROI box is around 2 × 2 mm, which might lead to inappropriate ROI placement on the lesion. Third, for VTIQ imaging, it was hard to completely avoid the calcification areas when the calcifications were diffused in the lesion, which might lead to bias since calcification in a lesion might increase tissue stiffness. Final, to confirm the factors associated with axillary LNM, a prospective, multicenter study with larger sample size is required in the future.

## Conclusion

In conclusion, lesion size ≥20 mm, taller than wide shape, VTI score ≥5, histological grade III, and positive C-erbB-2 are risk factors for axillary LNM. The risk model developed in the study could predict the risk of axillary LNM in patients with breast IDCs and thus might be helpful to avoid unnecessary ALND.
